# Supplementary material for: Development and psychometric validation of a scoring questionnaire to assess healthy lifestyles among adolescents in Catalonia
Source: BMC Public Health. 2016 Jan 28;16:89. doi: 10.1186/s12889-016-2778-6 (PMC4731967; doi:10.1186/s12889-016-2778-6)
Supplement: Supplementary file 1 — Non-validated English version from VISA-TEEN. (PDF 264 kb) [file 12889_2016_2778_MOESM1_ESM.pdf]

This is a non-validated English version from VISA-TEEN. Validated Spanish version with final format is attached in another file name *VISA-TEEN Spanish version*

Socio-demographic questions:
Month and year of bird:
Are you a: Boy / Girl
In what country were you born?
In what country was your father born?
In what country was your mother born?
What's your high?
What's your weight?

Q1.- Put in order from 1 to 6 the following food types. Number one means most frequency and number 6 means less frequency (not allowed to repeat numbers)
Fruits and vegetables
Red meats (pork, beef, …)
Bread, pasta, cereals, rice, potatoes, …
Butter, sweets, …
Chicken, fish, eggs, …
Milk, cheese, …

Q2.- From the following alcoholic drinks, indicate your consumption. From Monday to Thursday (beer, wine and champagne). From Monday to Thursday (Gin, Ron, Wisky). From Friday to Sunday

Q3.- How much of moderate physical activity did you do last week daily? Consider moderate activity the one that allows you to talk but with difficulties when you are doing it. For example: walk with speed or go ~~by bicycle~~. biking in the city going to school, but not quickly.

Q4.- How much of intense physical activity did you do last week daily? Consider intense activity the one that it would be difficult to talk while you do the activity. For example: running, play soccer, basketball.

Q5.- How many hours per day do you spend to do the following activities?
On a day off / On a working day
- You are connected to internet to chat or relate to other people (social network)
- You are gamming with your computer, phone,)

Q6.- How many glasses of liquid do you drink daily? (from getting up until bed time)
Do not count soft drinks.
Less than 2
3 to 5
6 to 8
More than 8

Q7.- How many cans of soft drinks do you drink per week? (during school time?)
Less than 2
3 to 5
6 to 8
More than 8

Q8.- How many cigarettes do you smoke per day?
Monday to Thursday / Friday to Sunday
None
From 1 to 10
From 11 to 20
From 21 to 30
More than 30

Q9.- Consumption of other illicit drugs. Think of the last 12 months and answer how many times you have consumed it.
Joints / another drugs
More than once per month
Less than once per month
Never

Q10.- Think of the last month, how many hours have you slept per night if you had class next day. (hours that you spend sleeping and not only in bed).
More than 9 hours
8-9
7-8
6-7
5-6
Less than 5 hours

Q11.- Think of any normal day, answer the following questions:
How many times do you brush your teeth?
How many times do you wash your hands with soap?
Never
I do not wash them daily
Once per day / Daily
Twice per day
Three times per day
More than three times per day
