# Supplementary material for: Development and psychometric validation of a scoring questionnaire to assess healthy lifestyles among adolescents in Catalonia
Source: BMC Public Health. 2016 Jan 28;16:89. doi: 10.1186/s12889-016-2778-6 (PMC4731967; doi:10.1186/s12889-016-2778-6)
Supplement: Supplementary file 2 — VISA-TEEN Questionnaire. (PDF 1462 kb) [file 12889_2016_2778_MOESM2_ESM.pdf]

VISA-TEEN

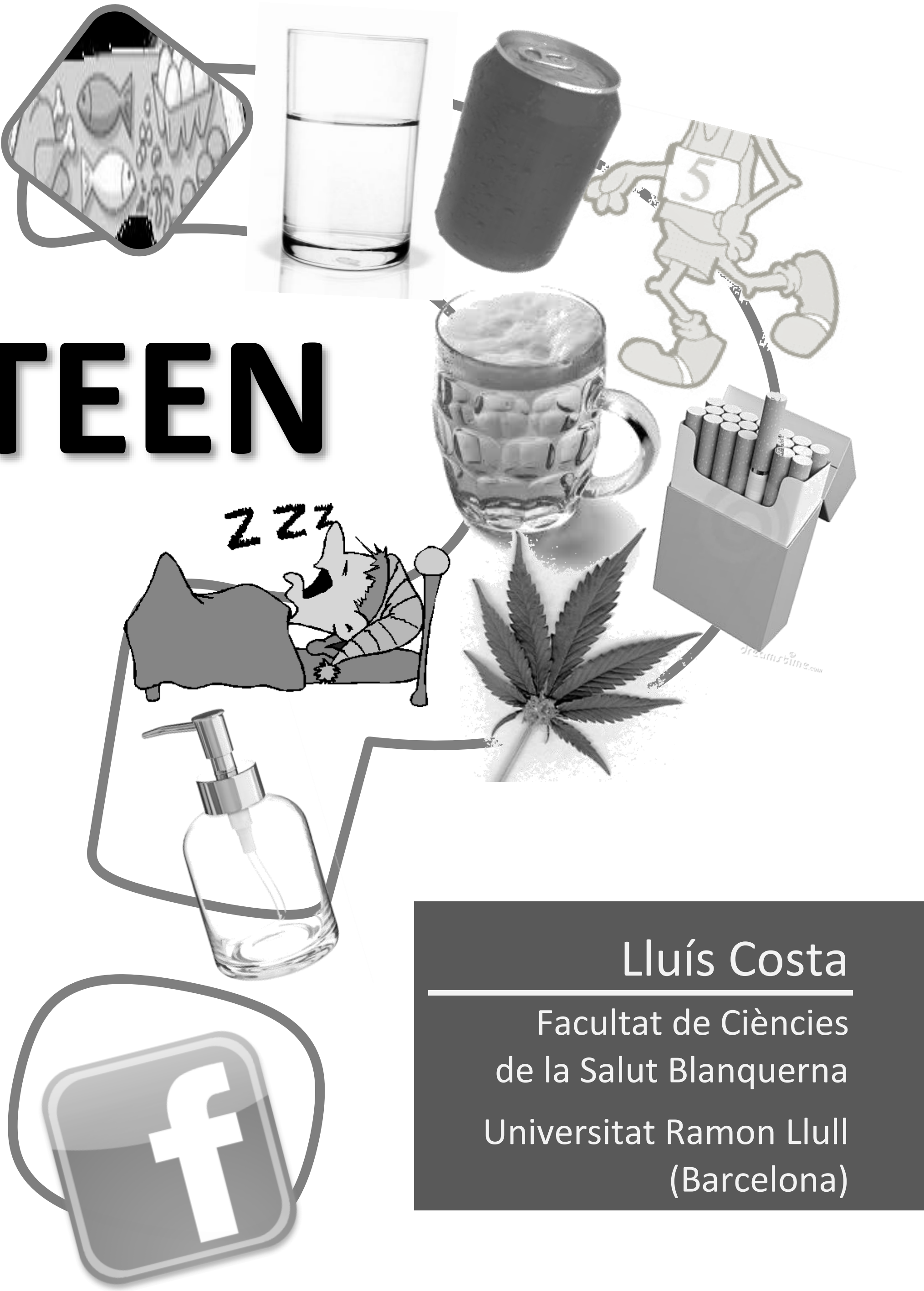

Lluís Costa

Facultat de Ciències
de la Salut Blanquerna

Universitat Ramon Llull
(Barcelona)

Cuestionario de estilo de VIda SAludable en adolescentes

Hola,

A continuación encontrarás una serie de cuestiones referentes a tu estilo de vida. El cuestionario es anónimo, y por lo tanto **puedes responder con toda sinceridad**.

Se trata de responder **lo que haces de verdad**, NO lo que crees que es correcto, o que "queda bien".

Lee bien las preguntas antes de responder. Verás que hay preguntas en las que tendrás que poner un número, y otras en las que sólo tienes que marcar con una **X** la opción que elijas; por favor, no dejes ninguna pregunta en blanco. Si en alguna pregunta no encuentras la opción de respuesta que tú pondrías, marca la que más se acerca.

¡Muchas gracias por tu colaboración!

____

Escribe aquí tu **mes** y **año** de nacimiento

mes

año

Eres

un chico

una chica

¿En qué **país** naciste **?**

¿En qué **país** nacieron tus padres **?**

madre

padre

¿Cuánto **mides?**

cm

¿Cuánto **pesas?**

kg

Las siguientes preguntas (desde la 1 hasta la 5),

las has de responder poniendo

un número

1

Ordena de 1 a 6

los siguientes grupos de alimentos

1 es el que comes con **más** frecuencia y
6 el que comes con **menos** frecuencia
[no puede haber números repetidos]

Pon aquí el número de orden

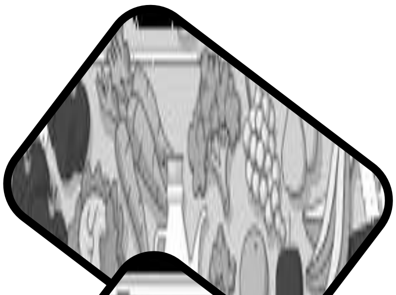

frutas, verdura, …

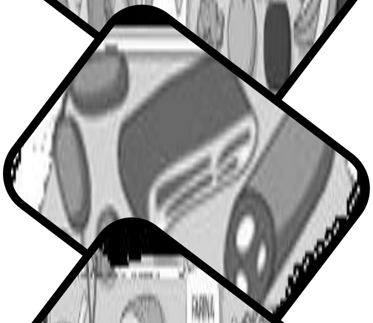

carnes rojas (cerdo, ternera), embutidos, …

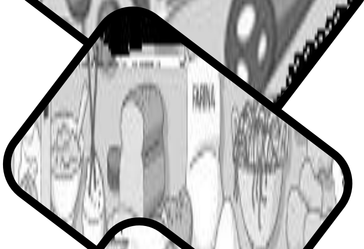

pan, pasta, cereales, arroz, patatas, …

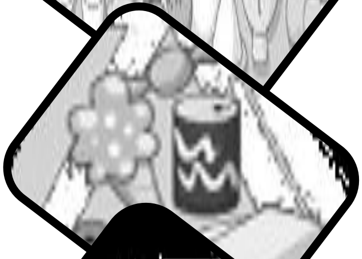

dulces, mantequilla, …

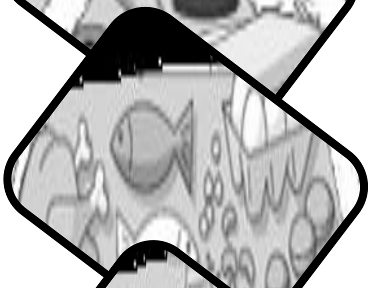

pollo, pescado, huevos, …

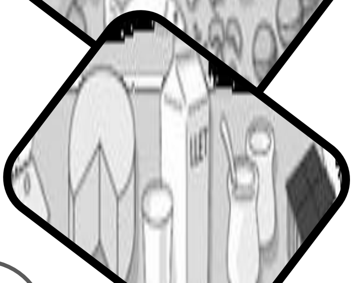

lácteos: yogurts, leche, queso, …

2 De las siguientes **bebidas alcohólicas**

indica la cantidad que consumes

[la cantidad es el número resultante de sumar todas las copas, jarras, vasos, *cubatas*, que hayan tomado en el período indicado]

Pon aquí el número.
Si no bebes nada,
pon un 0

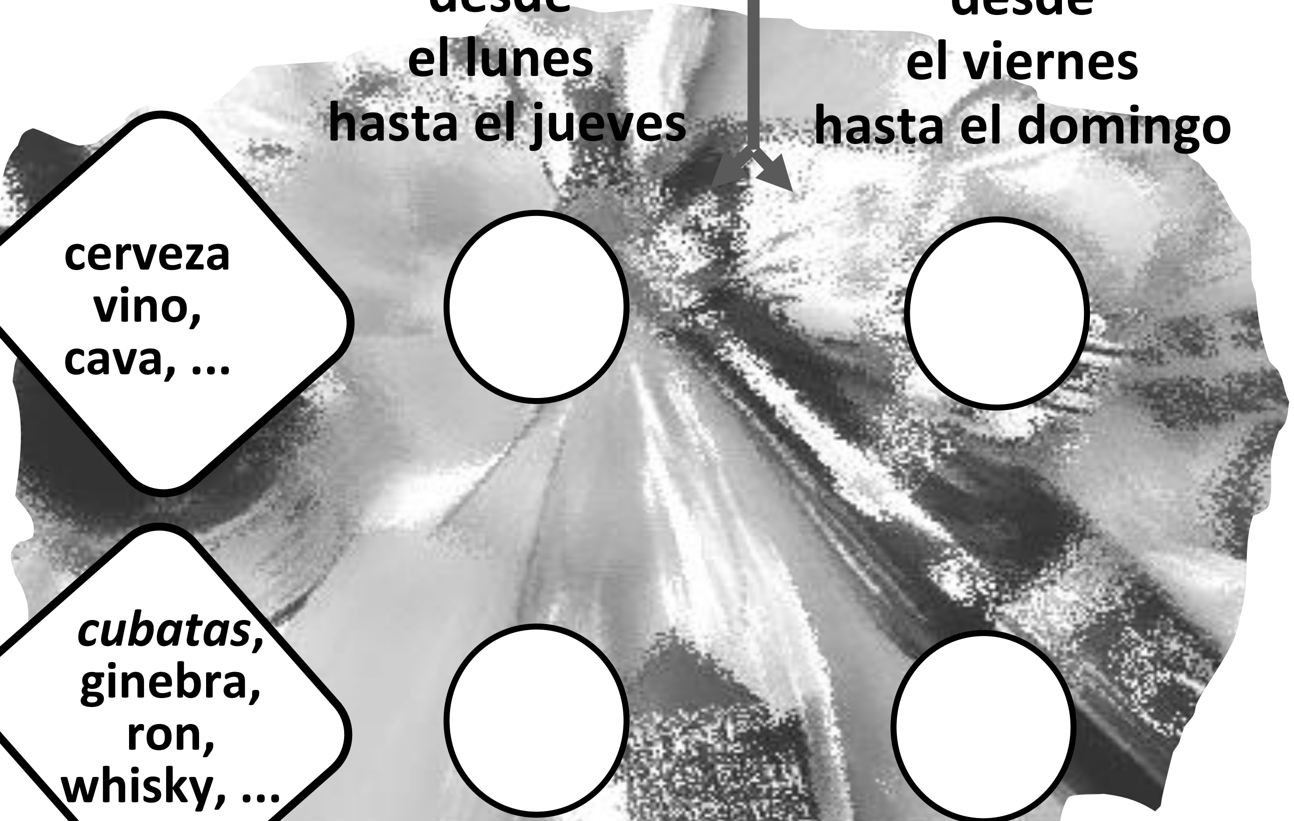

	desde el lunes hasta el jueves	desde el viernes hasta el domingo
cerveza vino, cava, ...		
cubatas, ginebra, ron, whisky, ...		

3

¿Cuánta actividad física **moderada** has hecho cada día de la semana pasada?

**es la actividad que te permite hablar,
pero con cierta dificultad,
cuando la realizas**

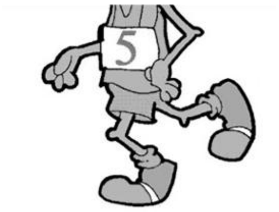

por ejemplo:

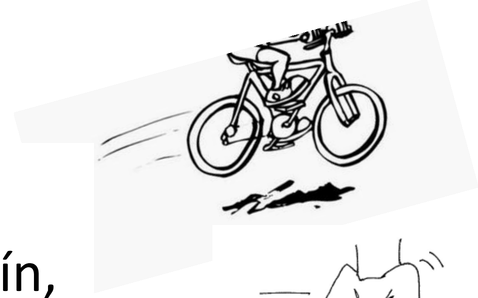

Andar deprisa o correr "al trote",
pasear en bicicleta o en monopatín,
jugar a voleibol, …

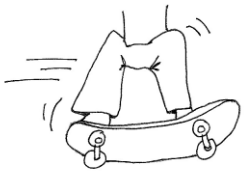

[cuéntalo en medias horas: **0'5**, **1**, **1'5**, **2**, …]

[pon el número en el círculo correspondiente a cada día]

**lu**	**ma**	**mi**	**ju**	**vi**	**sa**	**do**	**total**

4

¿Cuánta actividad física **intensa** has hecho cada día de la semana pasada?

es la actividad que, mientras la realizas, te costaría hablar seguido

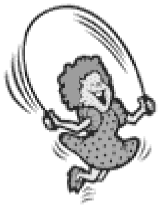

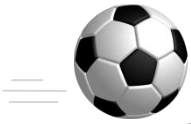

por ejemplo:

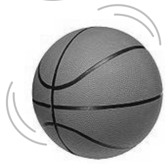

Correr deprisa, jugar al fútbol
o al básquet, saltar a la comba, ...

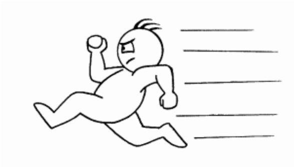

[cuéntalo en medias horas: **0’5**, **1**, **1’5**, **2**, …]

[pon el número en el círculo correspondiente a cada día]

**lu**	**ma**	**mi**	**ju**	**vi**	**sa**	**do**	**total**
○	○	○	○	○	○	○	●

5 ¿Cuántas **horas cada día**

Estás conectado a Internet (ordenador o móvil) para **chatear o relacionarte** con otras personas (redes sociales, msn, etc...)?

Estás jugando con el **ordenador, móvil, consola**, **juegos online**, o conectado a Internet para **entretenerte**? **(no** para chatear ni relacionarte a través de redes sociales**)**

[cuéntalo en medias horas: **0’5**, **1**, **1’5**, **2**, …]

[pon el número en el círculo correspondiente a cada día]

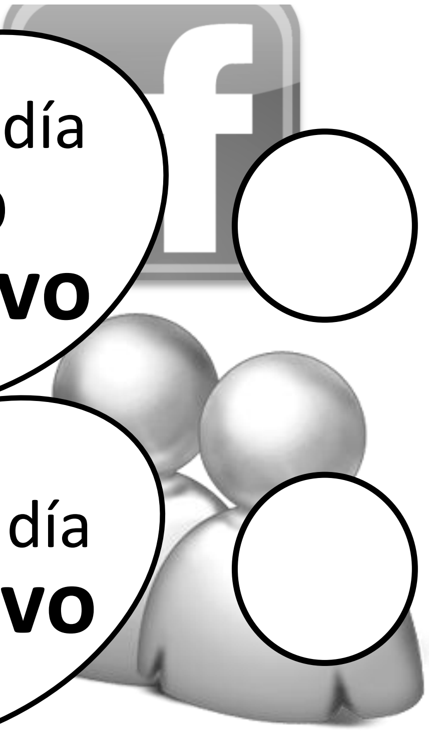

en un día **no festivo**

en un día **festivo**

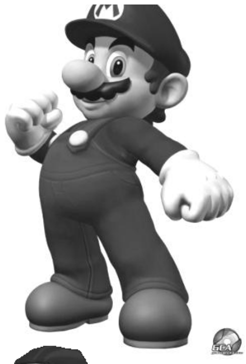

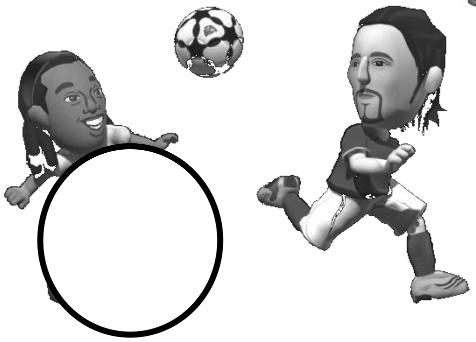

Las siguientes preguntas (desde la 6 hasta el final),

las has de responder marcando con

una cruz

la opción que selecciones

6

¿Cuántos
vasos de líquido*?
bebes diariamente
[contando desde que te levantas hasta que vas a dormir]

Indícalo con una cruz donde corresponda

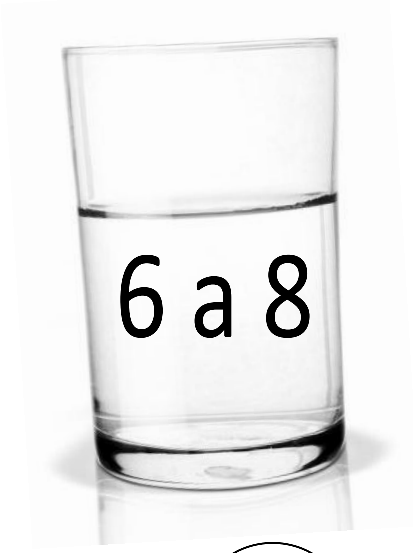

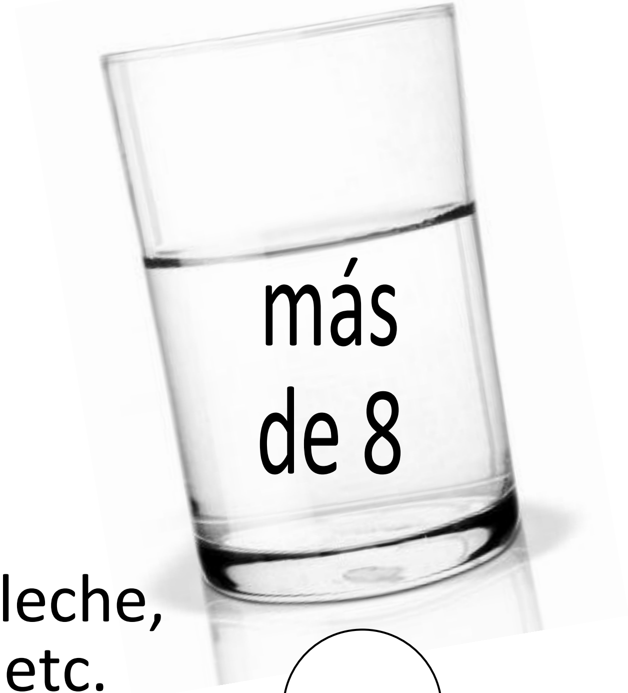

*Agua, leche, zumos, etc. **NO** cuentes los refrescos con gas

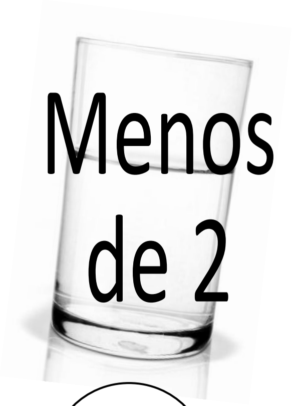

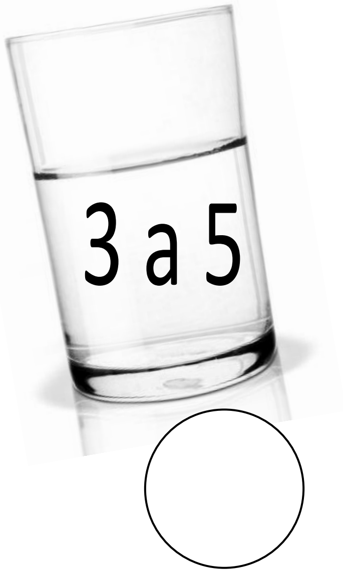

7

Cuántas

latas de refresco con gas*

bebes en toda una semana ?

[durante el curso escolar]

Indícalo con una cruz donde corresponda

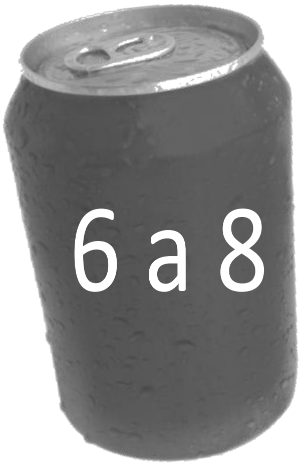

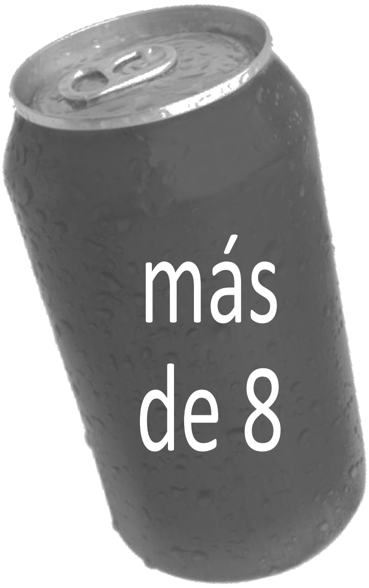

*Cola, naranjada, bebidas energéticas con gas, …

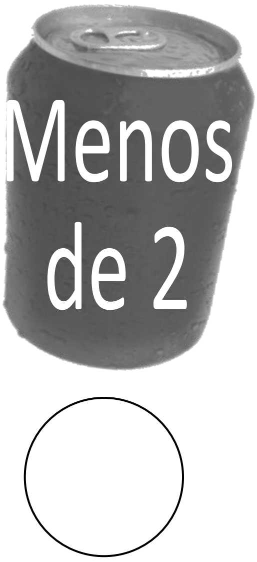

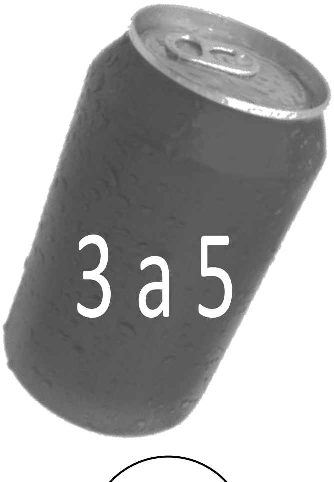

8

¿Cuántos **cigarrillos** fumas cada día?

Indícalo con una cruz donde corresponda

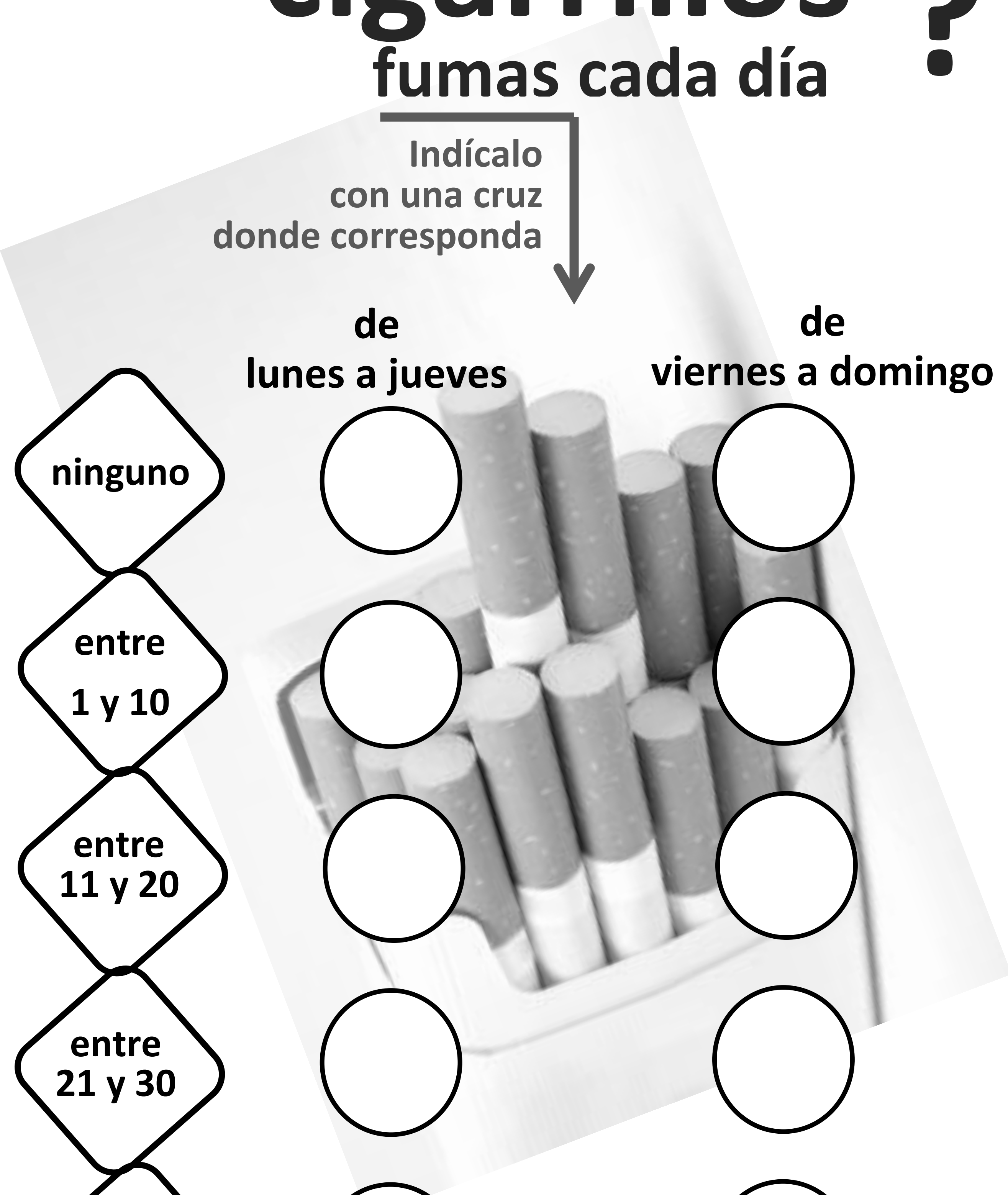

	de lunes a jueves	de viernes a domingo
ninguno	○	○
entre 1 y 10	○	○
entre 11 y 20	○	○
entre 21 y 30	○	○
más de 30	○	○

9

Consumo
de otras drogas

piensa en los últimos 12 meses, y responde cuántas veces has consumido...

Indícalo
con una cruz
donde corresponda

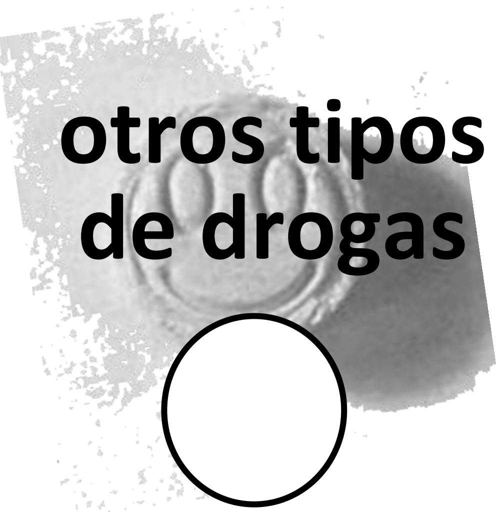

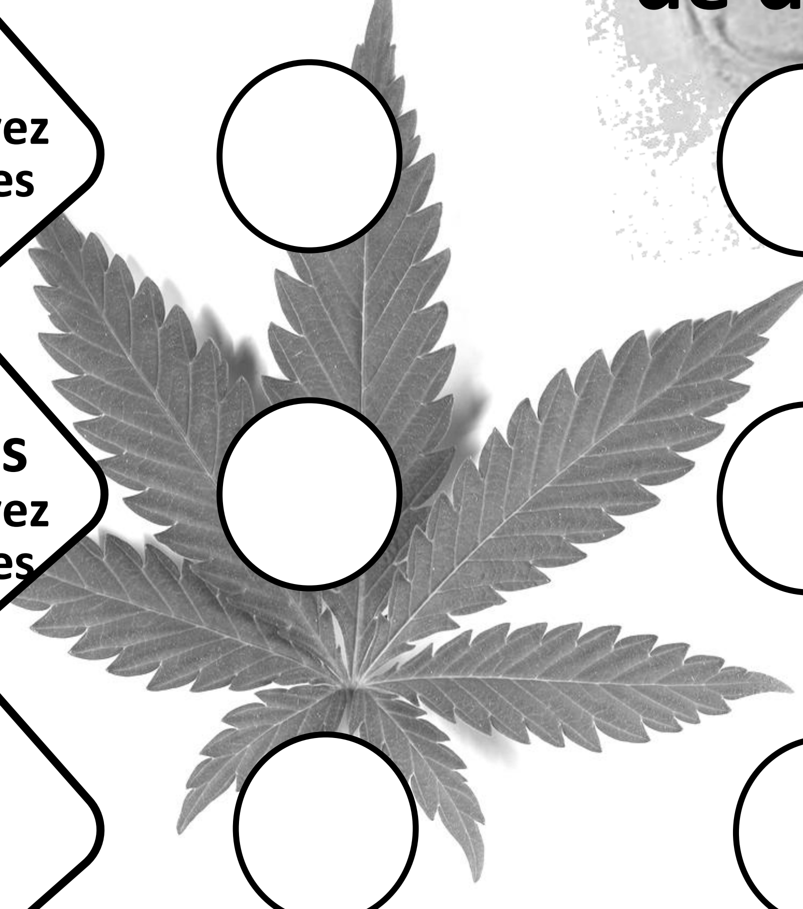

	porros	otros tipos de drogas
**más** de una vez cada mes	◯	◯
**menos** de una vez cada mes	◯	◯
nunca	◯	◯

10

Pensando en el último mes, cuántas horas **has dormido** cada noche, si al día siguiente tenías clase?

[horas que realmente duermes, no horas que pasas en la cama]

Indícalo con una cruz donde corresponda

- más de 9 ○
- 8-9 ○
- 7-8 ○
- 6-7 ○
- 5-6 ○
- menos de 5 ○

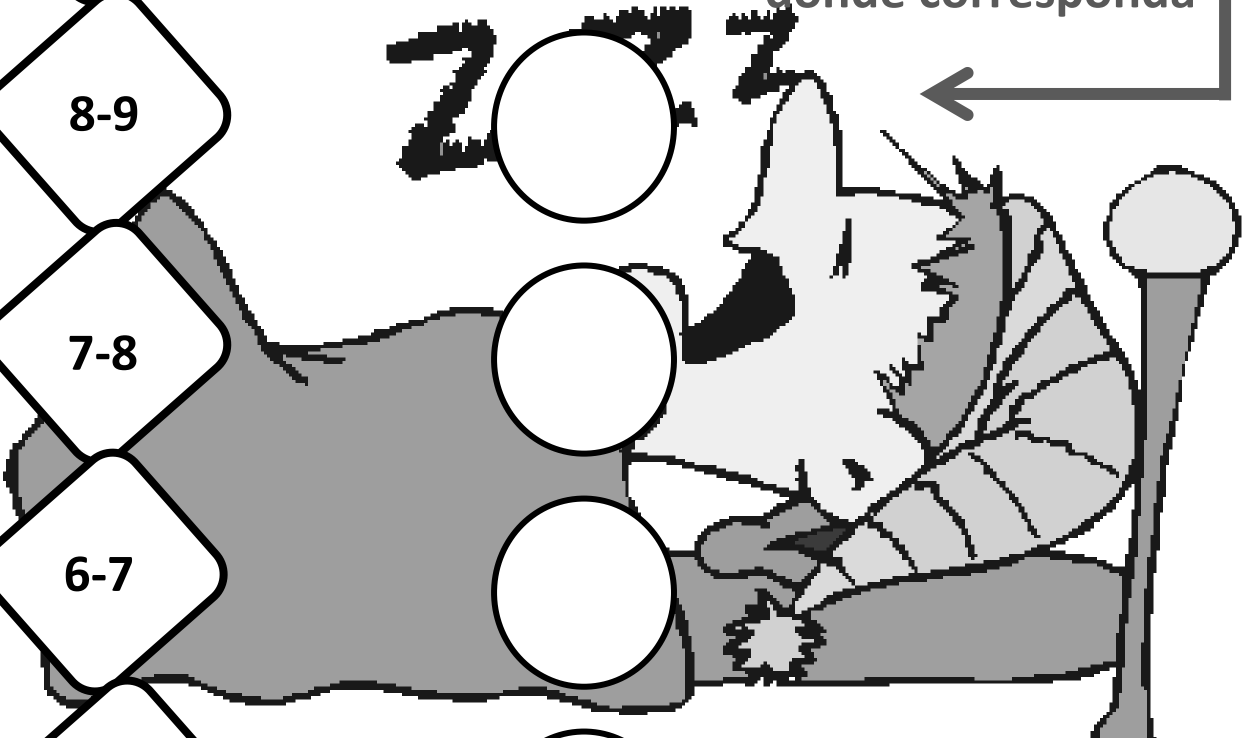

11 Pensando en un día cualquiera, responde

Indícalo con una cruz donde corresponda

	Cuántas veces te lavas los dientes	Cuántas veces te lavas las manos con agua y jabón
nunca	◯	◯
no me los lavo cada día	◯	◯
1 vez al día	◯	◯
2 veces al día	◯	◯
3 veces al día	◯	◯
más de 3 veces al día	◯	◯

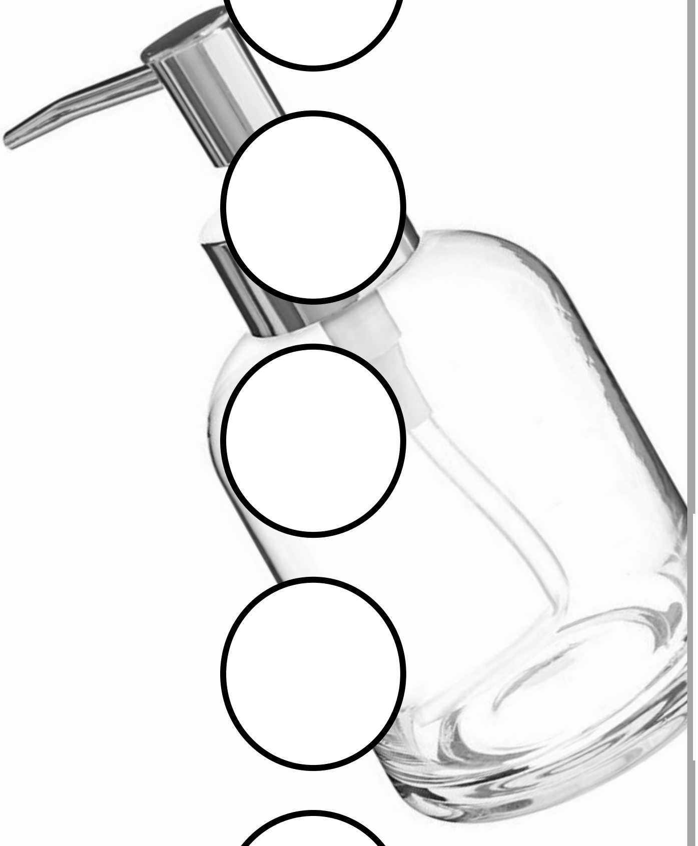

En general, ¿cómo dirías que es tu salud?

Excelente	Muy buena	Buena	Regular	Mala
◯	◯	◯	◯	◯
(1)	(2)	(3)	(4)	(5)

Durante los últimos 12 meses, ¿cuántas veces saliste de vacaciones con tu familia?

Nunca	Una vez	Dos veces	Más de dos veces
◯	◯	◯	◯
(1)	(2)	(3)	(4)

¿Cuántos ordenadores tiene tu familia?

Ninguno	Uno	Dos	Más de dos
◯	◯	◯	◯
(1)	(2)	(3)	(4)

¿Tiene tu familia coche propio o furgoneta?

No	Sí, uno	Sí, más de uno
◯	◯	◯
(1)	(2)	(3)

¿Tienes dormitorio para ti solo?

Sí	No
◯	◯
(1)	(2)
